# Bacterial Diversity and Lactic Acid Bacteria with High Alcohol Tolerance in the Fermented Grains of Soy Sauce Aroma Type Baijiu in North China

**DOI:** 10.3390/foods11121794

**Published:** 2022-06-17

**Authors:** Jiali Wang, Chengshun Lu, Qiang Xu, Zhongyuan Li, Yajian Song, Sa Zhou, Tongcun Zhang, Xuegang Luo

**Affiliations:** Key Laboratory of Industrial Fermentation Microbiology of the Ministry of Education, College of Biotechnology, Tianjin University of Science and Technology, Tianjin 300457, China; wangjiali816@163.com (J.W.); lcs19970305@163.com (C.L.); xq18228089685@163.com (Q.X.); lizhongyuan@tust.edu.cn (Z.L.); songyajian@tust.edu.cn (Y.S.); zhousa@tust.edu.cn (S.Z.); tony@tust.edu.cn (T.Z.)

**Keywords:** soy sauce aroma type baijiu, fermented grains, bacterial diversity, lactic acid bacteria, alcohol tolerance

## Abstract

Soy sauce aroma type baijiu (also known as Maotai-flavor baijiu) is one of the most popular types of baijiu in China. Traditionally, it is mainly produced in Southwest China. However, in recent decades, some other regions in China have also been able to produce high-quality soy sauce aroma type baijiu, but their microbial flora characteristics during fermentation are still unclear. Here, the bacterial microbial community structure of fermented grains in different rounds of Lutaichun soy sauce aroma type baijiu produced in North China was studied by high-throughput sequencing technology, and the potential probiotics strains with good characteristics (alcohol tolerance, etc.) were screened. The results showed that lactic acid bacteria were the main bacteria in the process of baijiu fermentation. However, as the number of repeated fermentation rounds increased, the proportion of lactic acid bacteria decreased. Firmicutes (96.81%) were the main bacteria in baijiu fermentation at the phylum level, and *Lactobacillus* (66.50%) were the main bacteria at the genus level. Finally, two strains with high resistance to alcohol stress, *Lactiplantibacillus plantarum* LTJ12 and *Pediococcus acidilactici* LTJ28, were screened from 48 strains of lactic acid bacteria in the fermented grains. The survival rates of *L. plantarum* LTJ12 and *P. acidilactici* LTJ28 under the 8% alcohol stress treatment were 59.01% and 55.50%, respectively. To the best of our knowledge, this study is the first to reveal the microbial succession of fermented grains in different rounds of soy sauce aroma type baijiu from North China, and has the benefit of explaining the deep molecular mechanism in the process of baijiu fermentation. In addition, the obtained lactic acid bacteria strains with high alcohol tolerance could be conducive to the development of new products such as active probiotic alcoholic beverages and may have important industrial development prospects also.

## 1. Introduction

Baijiu is the most popular alcoholic beverage in China, it has been made and drunk for more than 1000 years. Chinese baijiu is formed by solid-state fermentation with the interaction of various complex microorganisms and grains in a relatively open production environment [1]. The quality of baijiu is not only related to production technology, but also closely related to geographical environment, water quality, climate, choice of grain, and other factors [2]. Production in different regions and with different production processes leads to differences in the evolution of microbial flora in the process of fermentation, and therefore differences in the trace components and quality of the final distilled baijiu products [3,4].

At present, in general, Chinese baijiu can be mainly divided into 12 aroma types, such as soy sauce aroma type, strong aroma type, light aroma type, sesame aroma type, etc. However, the relationship between the aroma, flavor, quality of baijiu, and the microbial community characteristics of the fermentation process is still unclear. With the development of modern molecular biology, more and more techniques have been widely used in the study of microbial flora, such as restriction fragment length polymorphism (RFLP), fluorescence in situ hybridization (FISH), denaturing gradient gel electrophoresis (DGGE), real-time qPCR (RT-qPCR), high-throughput sequencing (HTS), etc. [5,6]. The application of these technologies provides technical support for the further study of baijiu fermentation. For sesame aroma type baijiu, Cui et al. [7] determined the changes in microbial community structure during its fermentation by 16S rDNA high-throughput sequencing combined with traditional methods of quantifying microorganisms, and found that cellulose bacteria, *Westermania,* and *Bacillus* were dominant in the early fermentation stage, and *Lactobacillus* was dominant in the middle and late fermentation stages, followed again by cellulose bacteria, *Westermania* and *Bacillus*. For strong aroma type baijiu, in the study by Zheng et al. [8], the spatial distributions of bacterial communities in the fermented grains from two particular factories (Jiannanchun and Fenggu) were investigated using culture-independent approaches, phospholipid fatty acid analysis (PLFA) and polymerase chain reaction/denaturing gel electrophoresis (PCR/DGGE). The results revealed that *Lactobacillaceae* was the dominant family in the top layers of the fermented grains from both Fenggu and Jiannanchun.

Lactic acid bacteria (LABs) are a group of bacteria that can use carbohydrates to produce lactic acid as a main metabolite. They are Gram-positive, catalase-negative, non-spore-bearing, non-motile, facultative anaerobic microorganisms and are considered as important probiotics for human health [9]. Any LABs suitable for industrial production should have good fermentation performance and strong tolerance, so that these microorganisms can survive during the process of production, in storage, and also in the gastrointestinal tract after being consumed by humans [10,11]. Among the unfavorable environmental challenge factors, alcohol, as a bacteriostatic organic reagent, can cause harmful changes in the cell membrane permeability of bacteria [12,13]. High concentrations of alcohol inhibit the growth and metabolism of LABs, block the intracellular transport system of glucose and amino acids, alter cell membrane properties, and affect membrane enzyme activity [14]. Therefore, it is extremely important to screen potential probiotic strains with strong tolerance to alcohol in the food and medicine industry.

The microbial flora of baijiu fermentation also provides a huge resource library for the discovery of new functional bacteria strains. Among these, as one type of important component during the baijiu fermentation process, the type, quantity and dynamic changes of LABs have an important impact on the aroma, flavor and quality of baijiu [2]. The main product of LAB is lactic acid, which can reduce the irritation but improve the mellow flavor of baijiu. LAB can also produce a small amount of acetic acid through heteromorphic fermentation, and acetic acid can affect the formation of baijiu flavor [15,16]. In addition, ethyl lactate produced by the reaction of lactic acid with ethanol can increase the sweetness and thickness of baijiu [1]. Finally, the organic acids produced by LABs can reduce the pH of the whole fermentation system and affect the growth and metabolism of other microorganisms in the microbial flora during baijiu fermentation [17,18]. Therefore, more and more researchers have begun to pay attention to LAB in the process of baijiu production.

As is well known, soy sauce aroma type baijiu is mainly produced in southwestern China (especially in Guizhou and Sichuan provinces), in locations such as Maotai (hence the name of Maotai-flavor baijiu) and Langjiu. However, in recent decades, some other regions in China have also been able to produce high-quality soy sauce aroma type baijiu, but their microbial flora characteristics during fermentation are still completely unclear. In this study, the microbial composition of bacteria from different rounds of fermented grains of soy sauce aroma type baijiu in northern China was analyzed using high-throughput sequencing technology to reveal the microbial succession and mechanism during the fermentation process of northern soy sauce aroma type baijiu. In addition, LABs with good characteristics (alcohol tolerance, etc.) were isolated, identified and screened using traditional isolation and culture methods. This investigation sought to lay some important foundations for future research into the production of soy sauce aroma type baijiu in northern China with the possibility of obtaining strains with good technological properties with possible uses as fermentation starters and in industrial processes, as well as the probiotics activity potential of some strains.

## 2. Materials and Methods

### 2.1. Sample Collection

The samples of fermented grains were taken from Tianjin Lutaichun Brewing Co., Ltd. Samples were taken from the 1st, 4th and 7th rounds of the same batch of fermented grains at the end of fermentation in the cellar with three replicates. A total of nine samples were aseptically treated and sealed and stored in an ultralow temperature refrigerator at −80 °C.

### 2.2. Bacterial Microbial Diversity Analysis of Fermented Grains

Microbial DNA was extracted from samples of fermented grains using the E.Z.N.A.^®^ soil DNA Kit (Omega Bio-tek, Norcross, GA, USA) according to manufacturer’s protocols. The final DNA concentration and purification were determined by NanoDrop 2000 UV-vis spectrophotometer (Thermo Scientific, Wilmington, USA), and DNA quality was checked by 1% agarose gel electrophoresis. The V3-V4 hypervariable regions of the bacteria 16S rRNA gene were amplified with primers 338F (5′-ACTCCTACGGGAGGCAGCAG-3′) and 806R (5′-GGACTACHVGGGTWTCTAAT-3′) by thermocycler PCR system (GeneAmp 9700, ABI, Los Angeles, CA, USA). The resulting PCR products were extracted from a 2% agarose gel and further purified using the AxyPrep DNA Gel Extraction Kit (Axygen Biosciences, Union City, CA, USA) and quantified using QuantiFluor™-ST (Promega, Madison, WI, USA) according to the manufacturer’s protocol. Purified amplicons were pooled in equimolar amounts and paired-end sequenced (2 × 300) on an Illumina MiSeq platform (Illumina, San Diego, CA, USA) according to the standard protocols by Majorbio Bio-Pharm Technology Co. Ltd. (Shanghai, China). Raw fastq files were quality filtered by Trimmomatic and merged by FLASH with the following criteria: (i) the reads were truncated at any site receiving an average quality score <20 over a 50 bp sliding window; (ii) sequences whose overlap was longer than 10 bp were merged according to their overlap with a mismatch of no more than 2 bp; (iii) sequences of each sample were separated according to barcodes (exactly matching) and primers (allowing 2 nucleotide mismatches), and reads containing ambiguous bases were removed. Operational taxonomic units (OTUs) were clustered with 97% similarity cutoff using UPARSE (version 7.1 http://drive5.com/uparse/, accessed on: 28 September 2020) with a novel ‘greedy’ algorithm that performs chimera filtering and OTU clustering simultaneously. The taxonomy of each 16S rRNA gene sequence was analyzed by RDP Classifier algorithm (http://rdp.cme.msu.edu/, accessed on: 28 September 2020) against the Silva (SSU123) 16S rRNA database using a confidence threshold of 70%. These indices (including Shannon, Simpson, Ace and Chao) were derived from different algorithms to estimate OTU [19]. 

### 2.3. Isolation and Purification of Bacterial Strains

Samples of 25 g from each round of fermented grains were weighed, mixed with 225 mL of sterile saline, and serially diluted to the appropriate multiple. An amount of 100 μL was applied to MRS medium plates and incubated upside-down at 37 °C for 24 h. The growth of colonies was observed. Single colonies were picked and microscopically examined by Gram staining to observe the morphological characteristics of the cells, and Gram-positive bacteria were selected as the target strains. The selected strains were scribed on MRS plates and repeatedly purified until no other miscellaneous bacteria were present, and the status of different strains was recorded.

### 2.4. Identification of the Selected Strains by 16S rDNA Sequence Analysis

The genome of the strain was extracted using Solarbio^®^ Bacterial Genomic DNA Extraction Kit (Beijing Solarbio Co., Ltd., China) and used as a template for PCR amplification. PCR reactions were performed using 27F (5′-AGAGTTTGATCCTGGCTCAG-3′) and 1492R (5′-GGTTACCTTGTTACGACTT-3′) as upstream and downstream primers for amplification of 16S rDNA sequences to identify the strains. The amplified products were sent to GENEWIZ Biotechnology Co., Ltd. (Tianjin, China) for sequencing, and the successfully amplified PCR products were sequenced and spliced to obtain DNA sequences. Sequence homology was compared and analyzed using Basic Local Alignment Search Tool (BLAST) (www.ncbi.nlm.nih.gov/blast/, accessed on: 28 September 2020). The known strains with the highest similarity to them were selected and their similarity was analyzed. The phylogenetic tree was established by using Neighbor-Joining (NJ) in MEGA 5 software (Tamura, Peterson, Stecher, Nei, and Kumar 2011) [20].

### 2.5. Determination of Lactic Acid Bacteria Tolerance

The strains at the end of logarithmic growth were selected and added to MRS liquid medium with different alcohol concentrations (4%, 6%, 8%, 10%, 12%) at 2% inoculum (bacterial concentration was 10^8^ CFU/mL) and subjected to alcohol stress for 12 h at 37 °C, while the MRS medium culture group without alcohol was used as a negative control. The *OD_600 nm_* was measured after the stress to compare their growth status with the control.

The strains at the end of logarithmic growth were selected and added to MRS liquid medium with pH 3.0 at 2% inoculum (bacterial concentration was 10^8^ CFU/mL) and incubated for 4 h at 37 °C, while the regular pH MRS medium culture group (pH 6.2) was used as a negative control. The *OD_600nm_* was measured after the stress to compare their growth status with the control.

The strains at the end of logarithmic growth were selected and added to MRS liquid medium containing 0.3% bile salt at 2% inoculum (bacterial concentration was 10^8^ CFU/mL) and incubated for 4 h at 37 °C, while the regular MRS medium culture group was used as a negative control. The *OD_600nm_* was measured after the stress to compare their growth status with the control.

### 2.6. Determination of Adhesion Ability of Lactic Acid Bacteria

A 96-well plate was coated with rat tail tendon collagen, and 100 μL of the bacterial solution was added to each well and incubated at 37 °C for 1 h with the bacterial-free solution as control. The unadhered bacteria were washed off with PBS buffer, followed by 10 μL of MTT per well for 4 h. A total of 100 μL of dimethyl sulfoxide (DMSO) was then added per well to dissolve the crystals. The absorbance values of the PBS-rinsed LAB, unrinsed LAB and unadded blank control wells were measured at 570 nm to calculate their adhesion capacity.

## 3. Results and Discussion

### 3.1. Bacterial Microbial Diversity during Baijiu Fermentation

During the brewing process of Lutaichun baijiu, the first, fourth, and seventh rounds of fermented grains from the cellar were sampled, and the bacterial microbial composition of fermented grains samples from different rounds was analyzed using high-throughput sequencing, with the first round of fermented grains from the cellar (LTJP1C) representing the early round, the fourth round of fermented grains from the cellar (LTJP4C) representing the middle round, and the seventh round of fermented grains from the cellar (LTJP7C) representing the late round. The Shannon and Simpson indices reflect the diversity of sample microbial species. Generally speaking, a greater diversity of microbial species will exhibit a higher Shannon index and a lower Simpson index. In addition, Chao and ACE indices always correlate positively with the abundance of microbial numbers in the sample. Table 1 shows the results of the bacterial α-diversity of the samples. From the first to the fourth round, the Shannon index declined from 1.59 to 1.28, before peaking at 1.83 in the seventh round, while the Simpson index moved in the opposite direction. The Shannon and Simpson indices showed that the diversity of bacterial species decreased slightly from the first to the fourth round, presumably due to the extreme environmental factors, and to the increased levels of ethanol and lactic acid, which could have inhibited bacteria growth and thus reduce bacterial diversity. In contrast, by the seventh round, with the increased consumption of raw materials, lower availability of starch, and the reduction in ethanol content, the environmental stress became milder, resulting in the highest level of bacterial diversity. At the same time, the Chao index increased from 51.42 to 61.20 and the ACE index increased from 56.65 to 68.62 between the first and the seventh round, indicating that the abundance of bacteria increased with the increase in rounds.

**Figure 1:**
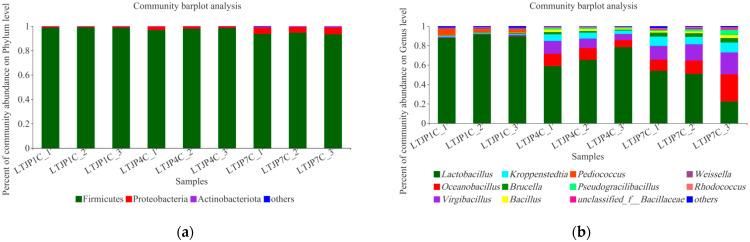
Dynamics of bacterial communities during baijiu fermentation at (**a**) phylum level and (**b**) genus level.

As shown in Figure 1a, the results of high-throughput sequencing analysis revealed that there were three major bacterial phyla in the first, fourth, and seventh rounds of fermented grains: Firmicutes (96.81%), Proteobacteria (2.65%), and Actinobacteria (0.47%). Among them, Firmicutes (mainly LABs) were the main bacteria in the baijiu fermentation, which gradually decreased from 98.88% to 93.83% from the first to the seventh round, while Firmicutes remained extremely high in all three rounds. Proteobacteria showed an opposite trend, rising from 0.81% to 5.20%, but with a low level maintained throughout. Figure 1b shows further dynamics at the level of bacterial genus. Overall, 11 major bacterial genera were detected: *Lactobacillus* (66.50%), *Oceanobacillus* (9.54%), *Virgibacillus* (9.46%), *Kroppenstedtia* (5.20%), *Brucella* (2.18%), *Bacillus* (1.53%), *Pediococcus* (1.50%), *Pseudogracilibacillus* (1.43%), unclassified_f_Bacillaceae (0.63%), *Weissella* (0.52%), and *Rhodococcus* (0.40%). Among them, *Lactobacillus* occupied an absolute dominant position. Since the environment was anaerobic during the fermentation in the cellar, *Lactobacillus* was more suited to grow in this environment, making *Lactobacillus* the absolute dominant bacteria. *Lactobacillus*, *Pediococcus*, and *Weissella* are all LABs, and LABs are the absolute dominant bacterial flora in the brewing process of baijiu, accounting for 68.52% of the total. At the species level, there was information for mainly eight LABs, including uncultured_*Lactobacillus*_sp._g_*Lactobacillus*, *Limosilactobacillus panis* (formerly *Lactobacillus panis*), *Lactobacillus acetotolerans*, *Lactobacillus amylovorus*, *Pediococcus acidilactici*, *Lentilactobacillus buchneri* (formerly *Lactobacillus buchneri*), *Lactiplantibacillus plantarum* (formerly *Lactobacillus plantarum*), and *Weissella paramesenteroides*. In a previous study, Hao et al. [21] used high-throughput sequencing technology to study the microbial community structure in the fermentation of Maotai-flavor baijiu (Guizhou Province, China) and found that LABs continuously increased and became the absolute dominant bacteria in the cellar fermentation. The results of this study were similar, but the difference was that Hao et al. only studied the microbial diversity in a single round of fermented grains, and so failed to reflect the microbial succession in the whole fermentation process of Maotai-flavor baijiu, making the value of their discovery was very limited. Our study was not limited to a single batch of fermentation, but studied the microbial diversity in the early, middle, and later stages of the whole baijiu fermentation process. Similarly, in the study of Wu et al. [16], LABs in solid-state fermentation stages were analyzed through high-throughput sequencing during the fermentation process. In total, 65 LAB species were identified in the fermented matrix. In addition, discrepancies were found to exist in the dominant LAB community structures during different fermentation stages, and strains of the *Lactobacillus* genus were found to be the most dominant LAB. However, the study by Wu et al. only verified the diversity of LABs in the fermentation process of Maotai-flavor baijiu (Guizhou Province, China), and did not confirm its proportion in the total bacteria community. This has been investigated in our study.

From the first round to the seventh round, the LABs (*Lactobacillus*, *Pediococcus*, and *Weissella*) decreased with the increase in rounds, from 95.53% to 42.49%, while *Bacillus* gradually increased. Among these LABs, *Lactobacillus*, including *Lactobacillus panis* (presently *Limosilactobacillus panis*), *Lactobacillus acetotolerans*, *Lactobacillus amylovorus*, *Lactobacillus buchneri* (presently *Lentilactobacillus buchneri*), *Lactobacillus plantarum* (presently *Lactiplantibacillus plantarum*), etc., gradually decreased from 89.47% at the first round of cellar discharge (LTJP1C) to 42.49% at the seventh round of cellar discharge (LTJP7C); *Pediococcus* (*Pediococcus acidilactici*) and *Weissella* (*Weissella paramesenteroides*) had higher levels only at the first round (LTJP1C) (4.49% and 1.57%, respectively). Li et al. [22] used high-throughput sequencing technology to sequence and analyze the microbial diversity of fermented grains from the production of soy sauce aroma type baijiu in Fujian Province of South China and found that *Lactobacillus* was the dominant bacteria in the fermented grains after cellar fermentation, but their proportion was greater at the end of cellar fermentation in the second round than in the middle of cellar fermentation in the third round, suggesting that *Lactobacillus* levels may have decreased with increasing rounds. The results of our study showed a similar result. However, Li et al. only studied the microbes of the fermented grains at the end of the second round of cellar fermentation and the middle of the third round of cellar fermentation. In our study, the fermented grains at the end of the first, fourth, and seventh rounds of cellar fermentation were taken, and the scope of the study covered the entire baijiu fermentation process. Therefore, the results of this study could reflect the dynamic microbial succession in the baijiu fermentation process more deeply. 

**Figure 2:**
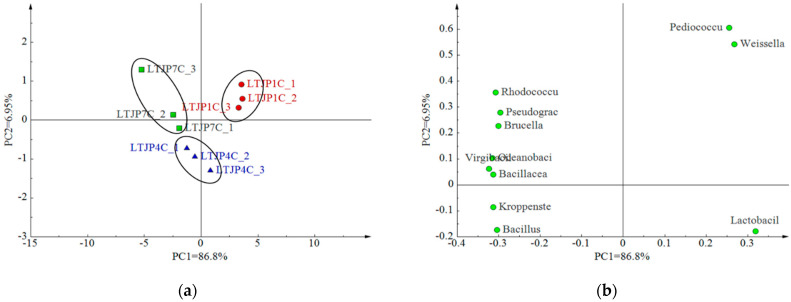
Principal component analysis (PCA) of bacterial communities and samples during baijiu fermentation. (**a**) Principal component analyses of samples during baijiu fermentation; (**b**) Loading plot for all bacterial communities during baijiu fermentation.

Based on the relative abundance of bacterial genera levels in the fermentation process of baijiu, multivariate data processing was performed using the software SICAM-P to analyze the distribution of bacteria among fermentation samples, and the results are shown in Figure 2. The horizontal coordinate of Figure 2 indicates the first principal component, which explained 86.8% of the variables; the vertical coordinate indicates the second principal component, which explained 6.95% of the variables, and the two principal components therefore explained a total of 93.75% of the variables, which could reflect the distribution of bacterial genera among the fermentation samples. The same rounds were clustered together—close to each other—in the score plot (Figure 2a). Combined with the loading plots (Figure 2b), the fermented grains from the first round of cellar fermentation (LTJP1C) and those from the fourth round of cellar fermentation (LTJP4C) were more correlated with *Lactobacillus*; the fermented grains from the first round of cellar fermentation (LTJP1C) were closely related to *Pediococcus* and *Weissella*.

In a similar way to Maotai, Lutaichun soy sauce aroma type baijiu analyzed in the present study also uses solid-state brewing technology with waxy sorghum as raw material. However, there are a few differences between northern Chinese varieties of soy sauce aroma type baijiu and those produced in the south of the country. It is well known that, along with environmental factors, baijiu production depends on climate, topography and soil [19]. The brewing process of southern soy sauce aroma type baijiu such as Maotai and Langjiu uses water from the Chishui River. In addition, southwestern China belongs to the subtropical humid monsoon climate zone, which is characterized by hot summers and warm winters, with a temperature range of 3–25 °C for most of the time each year, with an average of about 15 °C. Here, the number of cloudy days is generally more than 150 per year, and the annual relative humidity is above 70%. By contrast, Lutaichun soy sauce aroma type baijiu is produced in the Lutai Town of Tianjin. The water used for brewing Lutaichun baijiu is taken from the local Ordovician sweet spring, 800 m underground. Lutai Town is bordered by the Bohai Sea to the south and the Ji Canal to the north, and belongs to the temperate humid monsoon climate zone, in which the four seasons are more distinct than in Maotai. The temperature range in Lutai is −5–27 °C for most of the time each year, with an average of about 12 °C, while the annual sunshine duration can reach nearly 3000 h, and the annual relative humidity is above 60%. Obviously, such environmental differences will change the composition of the environmental microbial community, especially the dominant microbiota [19]. It is these differences between the north and the south that make the microbiota vary greatly in different regions, in the fermented grains of the same types of spirit. The essence of baijiu brewing is the process of microbial growth and metabolite accumulation, while the flavor and quality of baijiu rely on the synergistic effects between populations of microflora [3], which give baijiu its different flavors. 

In the study by Wu et al. [16], 52 LAB species were identified throughout the Maotai-flavor baijiu fermentation process, covering seven genera, with the most dominant genera being *Lactobacillus*, *Weissella*, *Pediococcus*. The order of abundance from high to low at species level was *Lactobacillus suebicus* (presently *Paucilactobacillus suebicus*), *Lactobacillus panis* (presently *Limosilactobacillus panis*), *Pediococcus acidilactici*, *Weissella paramesenteroides*, *Lactobacillus acetotolerans*, *Lactobacillus plantarum* (presently *Lactiplantibacillus plantarum*), *Lactobacillus buchneri* (presently *Lentilactobacillus buchneri*), *Lactobacillus amylovorus*. Our study found that the most dominant genera in the northern Lutaichun soy sauce aroma type baijiu were *Lactobacillus*, *Weissella*, and *Pediococcus*, while the order of abundance from high to low at species level was slightly different, being uncultured_*Lactobacillus*_sp._g_*Lactobacillus*, *Limosilactobacillus panis*, *Lactobacillus acetotolerans*, *Lactobacillus amylovorus*, *Pediococcus acidilactici*, *Lentilactobacillus buchneri*, *Lactiplantibacillus plantarum*, and *Weissella paramesenteroides*. Moreover, the succession of the dominant LAB varied slightly at different stages. In our study, although the proportion of LAB decreased with the increase in rounds, the variation of LABs of different species was also different. In the early rounds, except for uncultured_*Lactobacillus*_sp._g_*Lactobacillus* which was rarely identified, other LABs were the dominant LAB. By the middle and late rounds, uncultured_ *Lactobacillus*_sp._g_*Lactobacillus* became the dominant LAB, and all other LABs were at very low levels. The differences in LAB composition might play an important role in the different flavors of southern and northern soy sauce aroma type baijiu. The production of soy sauce aroma type baijiu is a spontaneous fermentation process that largely depends on regional microbial sources [6]. Therefore, exploring these sources could provide valuable information for understanding the complete microecology of baijiu fermentation and provide theoretical guidance for regulating the fermentation process of soy sauce aroma type baijiu.

In the brewing process of soy sauce aroma type baijiu, the main role of bacteria is to produce flavor and acid, and LAB is the dominant bacteria in cellar-fermented grains [22]. Studies have reported that, among LABs, *Lactobacillus*, *Leuconostoc*, *Lactococcus*, *Pediococcus*, and *Weissella* are regarded as the main functional genera in the fermentation process of Chinese baijiu [2]. *Lactobacillus*—as the core functional microorganism—is able to produce lactic acid, ethanol, and acetic acid through heterolactic fermentation to increase acidity [23]. *Lactobacillus* also regulates the acidity of fermented grains through lactic acid metabolism, thus inhibiting the growth of other miscellaneous bacteria [24,25]. In contrast, the higher acidity of soy sauce aroma type baijiu is reflected in a higher content of organic acids in the baijiu, which plays a buffering and coordinating role in and is one of the basic elements in determining the aroma and style of the baijiu, while the ethyl esters composed of organic acids and ethanol are the main aromatic substances of baijiu, and the abundant ethyl esters give the baijiu its rich and elegant aroma [6,22]. It can be seen that LABs play an important role in the fermentation of soy sauce aroma type baijiu, and the brewing process of soy sauce aroma type baijiu is a complex microecological fermentation system. The dynamic changes of its microbial succession need to be further studied.

### 3.2. Isolation and Identification of LAB in the Baijiu Fermentation Process

The fermentation process of baijiu is accompanied by special extreme environments such as high temperature, high acidity, and high ethanol [24]. Pit fermentation is the main stage of alcohol accumulation in the production of soy sauce aroma type baijiu, and during this process, LAB becomes the dominant bacteria [21]. A total of 48 strains of lactic acid bacteria were obtained from the fermented grains after isolation and purification, and they were observed for colony and cell morphology. The LABs obtained by preliminary isolation and purification can be mainly classified into five categories according to their colony and cell morphology (data not shown).

**Figure 3:**
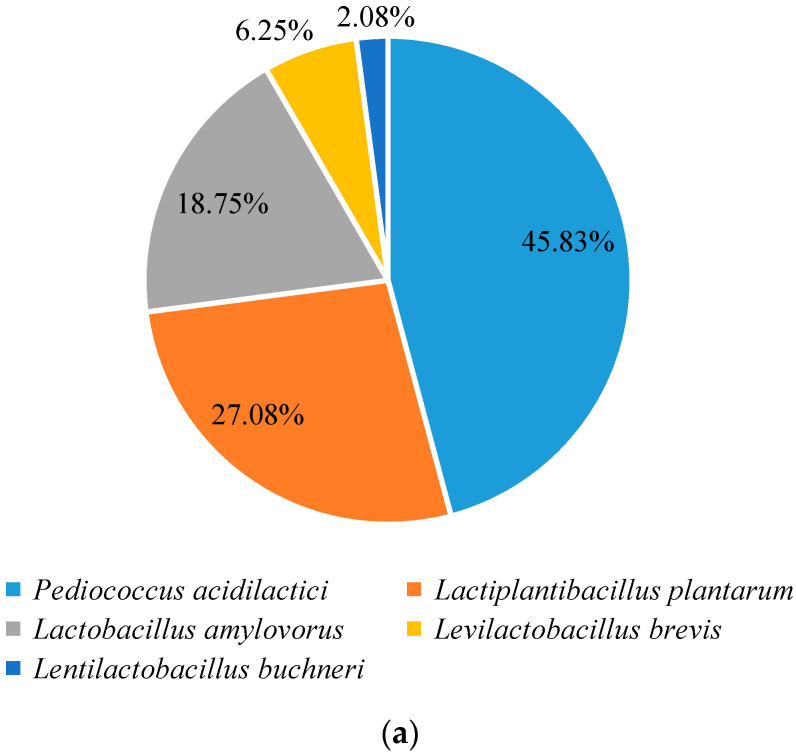

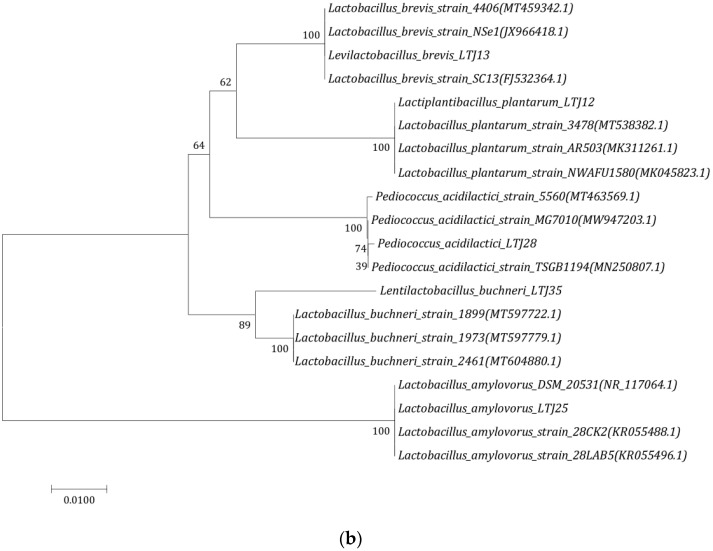
(**a**) Pie graph of species of lactic acid bacteria in the fermented grains of soy sauce aroma type baijiu and (**b**) phylogenetic tree based on 16S rDNA of typical strains.

The genomes of 48 strains of LAB were extracted, then amplified 16S rDNA sequences were sequenced and compared with the sequences in the NCBI database. Among the forty-eight obtained strains, twenty-two strains of *Pediococcus acidilactici*, thirteen strains of *Lactiplantibacillus plantarum* (formerly *Lactobacillus plantarum*), nine strains of *Lactobacillus amylovorus*, three strains of *Levilactobacillus brevis* (formerly *Lactobacillus brevis*), and one strain of *Lentilactobacillus buchneri* (formerly *Lactobacillus buchneri*) were found. The classified pie chart of LAB in fermented grains was shown in Figure 3a. Among the purified strains, *P. acidilactici*, *L. plantarum*, and *L. amylovorus* accounted for a larger proportion of the total number of strains in the fermented grains, which were 45.83%, 27.08%, and 18.75%, respectively. Meanwhile, the gene sequences of similar strains were downloaded to construct the phylogenetic tree, and the 16S rDNA sequence phylogenetic tree of five typical lactic acid bacteria was shown in Figure 3b.

### 3.3. Screening of Alcohol-Tolerant LABs

**Figure 4:**
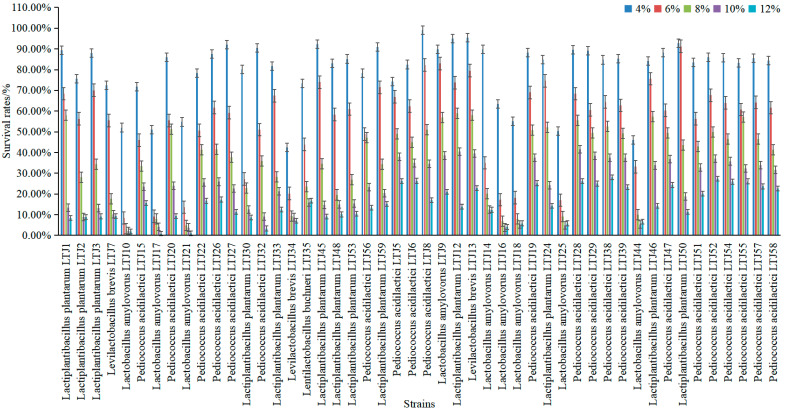
Effect of stress on the survival rates of strains during treatments with different concentrations of ethanol.

In the process of bacterial growth, high alcohol concentration affects the growth and activity of the bacteria to different degrees, thus affecting the quality of fermentation products. Therefore, it is very important to screen LABs with excellent resistance to alcohol stress. Forty-eight lactic acid bacteria strains were isolated and purified from fermented grains and treated with different alcohol concentrations to study the effect of different alcoholic conditions on the growth of the strains, and the results are shown in Figure 4. The alcohol tolerance of each strain isolated from the fermented grains showed a decreasing trend with the increase in alcohol concentration. Under the condition of 4% alcohol, all LABs grew with minimal inhibition, and the average alcohol tolerance rate of all strains was 79.86%. The growth of most *L. amylovorus* was somewhat inhibited when the alcohol content rose to 6%, while the growth of *L. plantarum* and *P. acidilactici* was less affected by alcohol, and the average alcohol tolerance rate of all strains was 55.17%. As the alcohol content increased to 8%, the average alcohol tolerance rate of all strains decreased to 36.67%. When the alcohol concentration finally reached 12%, alcohol tolerance rates ranged from 0.71% to 28.04%. However, and notably, *L. plantarum* and *P. acidilactici* showed better tolerance than other LAB species at each alcohol concentration. Indeed, *L. plantarum* LTJ12 and *P. acidilactici* LTJ28 were found to exhibit excellent alcohol tolerance at all alcohol levels. Under 4%, 6%, 8%, 10%, and 12% alcohol stress treatments, the survival rates of *L. plantarum* LTJ12 were 95.08%, 73.72%, 59.01%, 40.51%, and 13.81%, respectively, while the survival rates of *P. acidilactici* LTJ28 were 89.52%, 68.41%, 55.50%, 41.65%, and 26.21%, respectively. 

Reported studies have shown that 4% (*v*/*v*) ethanol stress can significantly inhibit the growth rate of LABs, while the growth rate approaches zero with 8% (*v*/*v*) ethanol. When the concentration of ethanol exceeds a certain value, bacteria cannot automatically maintain the stable state of cells, which may directly cause cell death [26]. Zhou et al. [27] isolated two alcohol-tolerant strains of *L. paracasei* from natural fruit and vegetable fermentation broth with good acid production, and these could tolerate 5% alcohol stress. In contrast, the strains screened in our study all exhibited survival rates above 40% under 10% alcohol stress, and still had high alcohol tolerance. Pradhan et al. [28] screened the probiotic and some of the functional properties of 37 LAB strains isolated from dry starters of the eastern Himalayas. A total of 86% of LAB strains present in the starters showed maximum tolerance at 10% ethanol. However, the two strains we screened not only had excellent tolerance at 10% alcohol, but also survived at still higher alcohol concentrations of 12%.

**Figure 5:**
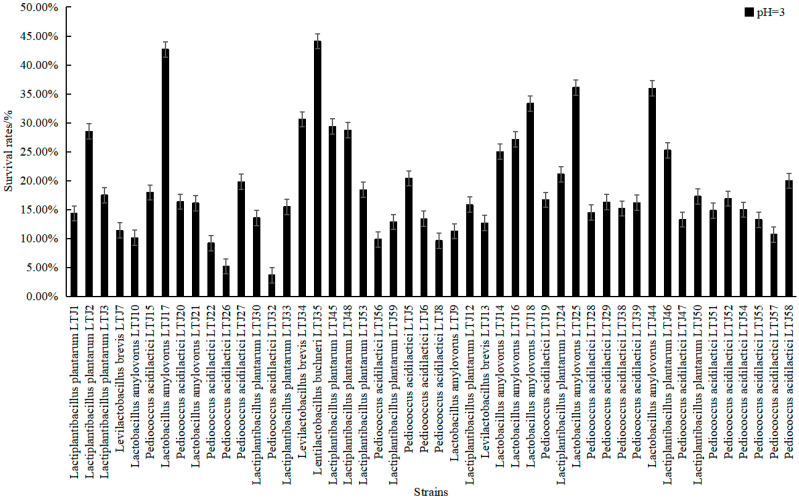
Determination of differences in acid resistance of strains.

In addition to alcohol stress, acid stress was also an important factor affecting the strains. Based on the determination of alcohol tolerance, the acid tolerance of 48 strains was also determined. As shown in Figure 5, the growth of most of the LABs was inhibited in an acidic environment of pH 3, and the average survival rate of all strains was 18.82%. *L. buchneri* LTJ35 had the strongest acid tolerance with a survival rate of 44.12%. Most of the *L. amylovorus* had strong acid tolerance. In the determination of alcohol and acid tolerance of strains, it was found that most of the strains with strong alcohol tolerance had weak acid tolerance. On the contrary, most of the strains with weak alcohol tolerance had strong acid tolerance. Among these, the acid tolerance ability of *L. plantarum* LTJ12 and *P. acidilactici* LTJ28, which both had stronger alcohol tolerance ability, were 15.87% and 14.49%, respectively. Bravo-Ferrada et al. [29] also conducted alcohol and acid tolerance experiments on *L. plantarum* isolates obtained from a Patagonian red wine, and obtained strains with better alcohol and acid resistance. However, due to the difference in experimental methods and data processing, it is impossible to determine whether their strains or our strains were better in this regard.

**Figure 6:**
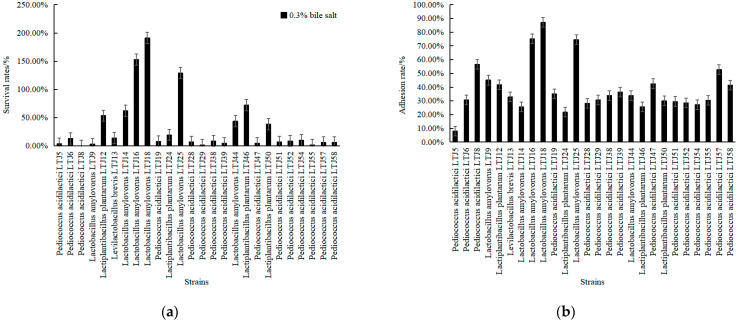
Determination of difference in (**a**) bile salt resistance and (**b**) adhesive ability to collagen of strains.

Bile salt tolerance and colonization ability are very important for the survival of probiotics in the host. A higher strain colonization capacity allows more strains to grow and multiply, and low survival rates in acid and bile salt environments mean that strains have difficulty surviving in the host, which is contrary to the definition of probiotics [20]. Combining alcohol tolerance and acid tolerance, 26 strains of LABs were selected to analyze their bile salt tolerance and adhesion ability. As shown in Figure 6, most of the *L. amylovorus* had strong bile salt tolerance. The survival rate of *L. plantarum* LTJ12 and *P. acidilactici* LTJ28 reached 53.38% and 6.89%, respectively, in the MRS medium containing 0.3% bile salts for 4 h, and the extracellular matrix adhesion rates were 41.84% and 28.18%, respectively. 

Traditionally, fermented foods have been a good source of new potential probiotics. LABs with good tolerance properties not only add health conditioning in food fermentation, but also enriched flavor and other beneficial effects. Probiotics, as live microbial food supplements, benefit the host by improving the intestinal microbial balance [30]. As is well known, a large number of studies have proved the safety and probiotic properties of *L. plantarum* and *P. acidilactici*, and they are on the list published in China of bacteria that can be used in food. *L. plantarum* strains have been marketed as starters; however, this species also includes strains with documented probiotic properties, such as *L. plantarum* WCFS1 and *L. plantarum* 299v [31]. Similarly, it has been reported that *P. acidilactici* is generally considered safe and possesses probiotic properties such as beneficial enzymatic activity [32], and it is widely used in food fermentation and as a starter for cheese and yogurt production [33]. Therefore, these results suggest that *L. plantarum* LTJ12 and *P. acidilactici* LTJ28 could have great potential for future development as probiotic strains.

## 4. Conclusions

The bacterial microbial community structure was studied by high-throughput sequencing. The results showed that LABs (mainly *Lactobacillus*) were the main bacteria in the fermentation process of soy sauce aroma type baijiu in northern China, but their levels decreased with increasing numbers of fermentation rounds. Among the new strains of LAB, isolated from fermented grains, two strains with high resistance to stress of alcohol, acid and bile salt and good extracellular matrix adhesion ability, *L. plantarum* LTJ12 and *P. acidilactici* LTJ28, were screened. To the best of our knowledge, this study is the first report that reveals the microbial succession of the fermented grains from the cellar of soy sauce aroma type baijiu produced in northern China, and which helps to explain the deep molecular mechanism of the fermentation process of baijiu, thus providing some theoretical guidance for the process control and improvement of traditional baijiu. In addition, the screening of lactic acid bacteria strains with high alcohol tolerance in the present study may also advance the possibility of obtaining strains with good technological properties with possible uses as fermentation starters and in industrial processes, as well as the probiotics activity potential of some strains.

## Figures and Tables

**Table 1:** Bacterial alpha diversity indices during baijiu fermentation.

Sampling Points	Samples	Shannon	Simpson	Ace	Chao
The first round out of the cellar (LTJP1C)	LTJP1C_1	1.67	0.28	61.89	57.50
	LTJP1C_2	1.47	0.35	58.43	48.75
	LTJP1C_3	1.64	0.31	49.63	48.00
	Average	1.59	0.31	56.65	51.42
The fourth round out of the cellar (LTJP4C)	LTJP4C_1	1.51	0.38	56.26	45.20
	LTJP4C_2	1.35	0.45	56.02	50.17
	LTJP4C_3	0.99	0.62	69.49	68.33
	Average	1.28	0.48	60.59	54.57
The seventh round out of the cellar (LTJP7C)	LTJP7C_1	1.70	0.33	81.98	80.60
	LTJP7C_2	1.70	0.30	61.12	45.00
	LTJP7C_3	2.10	0.15	62.77	58.00
	Average	1.83	0.26	68.62	61.20

## Data Availability

The data presented in this study are available within the article.
